# Colorectal cancer liver metastases organoids retain characteristics of original tumor and acquire chemotherapy resistance

**DOI:** 10.1016/j.scr.2018.01.016

**Published:** 2018-03

**Authors:** Jon N. Buzzelli, Djamila Ouaret, Graham Brown, Philip D. Allen, Ruth J. Muschel

**Affiliations:** a Old Road Research Campus Building, Department of Oncology, University of Oxford, Oxford, UK; b Cancer and Immunogenetics Laboratory, Weatherall Institute of Molecular Medicine, Department of Oncology, University of Oxford, John Radcliffe Hospital, Oxford OX3 9DS, UK

**Keywords:** Organoid, Liver metastasis, Colorectal cancer, Chemotherapy resistance

## Abstract

**Background:**

Colorectal cancer (CRC) liver metastasis is highly unfavorable for patient outcome and is a leading cause of cancer-related death. Pre-clinical research of CRC liver metastasis predominately utilizes CRC cell lines grown in tissue culture. Here, we demonstrate that CRC liver metastases organoids derived from human specimens recapitulate some aspects of human disease.

**Methods:**

Human CRC liver metastases pathological specimens were obtained following patient consent. Tumor disaggregates were plated and organoids were allowed to expand. CRC markers were identified by immunofluorescence. Stem cell genes were analysed by QPCR and flow cytometry. Response to drug therapy was quantified using time-lapse imaging and MATLAB analysis.

**Results:**

Organoids showed global expression of the epithelial marker, EpCAM and the adenocarcinoma marker, CEA CAM1. Flow cytometry analysis demonstrated that organoids express the stem cell surface markers CD24 and CD44. Finally, we demonstrated that CRC liver metastases organoids acquire chemotherapy resistance and can be utilized as surrogates for drug testing.

**Conclusion:**

These data demonstrate that CRC liver metastases organoids recapitulate some aspects of human disease and may provide an invaluable resource for investigating novel drug therapies, chemotherapy resistance and mechanism of metastasis.

## Highlights

• Human colorectal cancer liver metastases can be cultured as organoids using rapid dissociation.
• Organoids retain characteristics of human disease.
• Organoids acquire chemotherapy resistance.

## 1 Introduction

Colorectal cancer (CRC) is the 3rd most common cancer worldwide. Fatality is most commonly caused by metastasis, a multistep process which requires tumor cell dissemination and colonization of a foreign organ (Friedl and Wolf, 2003). Advantageous mutations which permit tumor cells to disseminate also contribute to chemotherapy resistance (Scheel and Weinberg, 2012). Furthermore, drug therapy in the metastatic setting provides little advantage due to the frequency of metastatic relapse and drug resistance (Wan et al., 2013, Yokobori et al., 2013).

Currently, pre-clinical investigation of metastasis relies on cell lines derived from CRC. While such studies have yielded invaluable insights into mechanisms which permit metastasis, these cell lines do not accurately represent all some aspects of advanced disease (Edwards et al., 2015, Marshall, 2014). Furthermore, novel drug therapies are commonly only tested on human-derived primary cultures. Collectively, this suggests that the development of human-derived cultures of CRC liver metastases would provide an invaluable resource for advancing our understanding of the mechanisms which permit tumor cell migration and invasion, and allow drug screening in cultures which recapitulate advanced stages of human disease. Here, we expand on previous work done on establishing CRC organoids (Ashley et al., 2014, Ashley et al., 2013, Fujii et al., 2016, Sato et al., 2011, Weeber et al., 2015). We demonstrate that human CRC liver metastases organoids can be rapidly cultured with mechanical dissociation, and these samples recapitulate some aspects of human disease. Furthermore, we show that these cultures can be utilized as surrogates for drug screening and acquire chemotherapy resistance.

## 2 Materials and methods

### 2.1 Human specimens and organoid culturing

Human CRC liver metastases pathological specimens were obtained from the Oxford Radcliffe Biobank following patient consent, institutional review and ethical approval. Pathological specimens were immediately placed in DMEM/F12 + GlutaMAX (ThermoFisher, USA, CAT #31331-028) containing 1% Penicillin/Streptomycin and 0.4% Ampicillin B. Pathological specimens were divided and embedded in Optical Cutting Temperature (OCT) for immunofluorescence, snap frozen for molecular analysis or transferred to fresh DMEM/F12 + GlutaMAX for tumor disaggregation as previously described (Ashley et al., 2014). Disaggregates were plated in a 96-well suspension plate (Sarstedt, GER, CAT #83.3924.500) or mixed with Matrigel Matrix (Corning, USA, CAT #354234) and plated into a 24-well suspension plate (Sarstedt, CAT #83.3922.500). For Matrigel mixed samples, 50 μL was added per well. DMEM/F12 + GlutaMAX containing StemPro, ROCKs inhibitor, R-Spondin-1 (RSPO-1), Noggin, WNT3A, Epithelial Growth Factor (EGF), Insulin-like Growth Factor 1 (IGF-1), Fibroblast Growth Factor 10 (FGF-10), Fibroblast Growth Factor basic (FGFβ) and Endothelin 3 (ET3) was added to the samples (Refer to Sup. Table 1 for concentrations). Media was changed 3 times/week and cultures were passaged every 1–2 weeks. Passaging was performed by incubating cultures in ice-cold PBS for 15 min, followed by 2 washes in ice-cold PBS. Cultures were then centrifuged and dissociated by pipetting. Culture disaggregates were then resuspended in PBS and counted under a light microscope. Approximately 50 crypt-like disaggregates were resuspended in Matrigel and plated in 4–8 wells of a 24-well suspension plate. DMEM/F12 + GlutaMAX containing all growth factors will be referred to as media with full supplementation. For all analysis and experiments, the passage number of cultures was between 8 and 16.

### 2.2 Hematoxylin and Eosin (H&E) staining and immunofluorescence

Organoid cultures were isolated from Matrigel and washed 3 times with ice cold PBS then immediately embedded in OCT freezing media and snap frozen in liquid nitrogen. 10 μm sections were collected and stored at − 30 °C. Prior to staining, sections were dried for 1 h at 37 °C. For H&E staining, slides were hydrated in graded ethanol solutions then placed in Heamatoxylin Harris (VMR Chemicals, USA, CAT #3519455) for 2 min, rinsed in tap water and differentiated in 1% hydrochloride in 70% ethanol for 30 s. Following differentiation, samples were washed in tap water then incubated in Eosin solution (Sigma, USA, CAT #HT110132) for 2 min before being dehydrated in graded ethanol. Slides were incubated in xylene before being mounted with Vecta Mount (Vector, CAT #H-5000). For immunofluorescence, slides were fixed in 4% paraformaldehyde and permeabilized with 0.1% Triton. Slides were blocked for 1 h at room temperature (RT) and primary antibodies were left on overnight at 4 °C. The next day, slides were washed and secondary antibodies were added for 1 h at RT in the dark. Slides were then stained with Hoechst (1 in 250, 5 mg/mL ThermoFisher, CAT #33342) for 10 min and mounted with ProLong Diamond Antifade Mountant (ThermoFisher, CAT #P36961). Immunofluorescence images were captured with a Leica DM6000 confocal microscope. Antibodies were used at the following concentrations; Rabbit anti-human Pan-Laminin: 1 in 100 (Sigma, CAT #AB11575), secondary: Donkey anti-rabbit 555: 1 in 250 (ThermoFisher, CAT #21428); Mouse anti-human CEACAM5 (Santa Cruz, CAT # SC-23928): 1 in 100, secondary: Goat anti-mouse 546: 1 in 250 (ThermoFisher, CAT #A21123); Mouse anti-human EpCAM (AUA1 antibody raised in house) (Ashley et al., 2014): 1 in 100, secondary: Goat anti-mouse 546: 1 in 250 (ThermoFisher); Rabbit anti-human Ki67: 1 in 100 (Vector Labs, CAT #VP-RM04), secondary: Goat anti-rabbit 488: 1 in 250 (ThermoFisher CAT #A11008); Rabbit anti-human MUC2: 1 in 100 (Santa Cruz; CAT #SC-15334), secondary: Donkey anti-rabbit 555: 1 in 250 (ThermoFisher, CAT #21428). To quantify EpCAM and MUC2 staining, a Pathologist identified regions of tumor tissue within CRC liver metastases pathological specimens, and the percentage of EpCAM and MUC2 positive staining was measured. For CRC liver metastases organoids, the percentage of EpCAM and MUC2 positive staining was measured for the total organoid section. Measurements were obtained using ImageJ.

### 2.3 Flow cytometry (FACs)

Organoid cultures were isolated from Matrigel and washed 3 times with ice cold PBS. To generate a single cell suspension, organoids were incubated in TrypLE (ThermoFisher CAT #12604021) for 30 min at 37 °C before being passed through a 70 μm Nylon cell strainer. Single cell suspensions were washed with PBS and then stained with EpCAM, CEA CAM1, CD24, CD44, CD133, CD166, CD31 and CD45 for 30 min in the dark on ice. Samples were washed with 1 mL of PBS then resuspended in 2% Fetal Bovine Serum and ran through a Fortessa Flow Cytometer (BD Bioscience, USA). Analysis was performed using FlowJo V10 Software. Refer to Sup. Table 2 for flow cytometry antibody information.

### 2.4 RNA extraction and quantitative PCR (QPCR) analysis

RNA was harvested using TRIzol reagent (Life Technologies). RNA (1 μg) was reverse transcribed using Moloney murine leukemia virus reverse transcriptase (Promega) primed with oligo (dT). Quantitative PCR (QPCR) primers were designed using PRIMER EXPRESS (Applied Biosystems). SYBR green chemistry was used with Rpl32 as the internal reference gene. The conditions were 95 °C for 10 min, 40 cycles of 95 °C for 30 s and 60 °C for 1 min (Stratagene Mx3005P). QPCR analysis was performed on 4 technical replicates for each group, and results were analysed using sequence detector software, relative fold differences were determined using the ΔΔCt method. Human Rpl32-forward: 5-CATCTCCTTCTCGGCATCA-3′; human Rpl32-reverse: 5-ACCCTGTTGTCAATGCCTC-3′. Human ALDH1-forward: 5-TGTTAGCTGATGCCGACTTG-3′; human ALDH1-reverse: 5-TTCTTAGCCCGCTCAA CACT-3′. Human PROX1-forward: 5-CAGATGGAGAAGTA CGCAC-3′; human PROX1-reverse: 5-CTACTCATGAAGCAGCTCTTG-3′. Human LGR5-forward: 5-AACAGTCCTGTGACTCAACTCAAG-3′; human LGR5-reverse: 5-TTAGAGACATGGGACAAATGCCAC-3′. Human ABCG2-forward: 5-GGCCTTGGGA TACTTTGAATC-3′; human ABCG2-reverse: 5′-CTACTCATGAAGCAGCTCTT G-3′. Human CDH1-forward 5′-AGCTTGCGGAAGTCAGTTCA-3′; human CDH1-reverse: 5′-CAGAAACGGAGGCCTGATGG-3′; human CEA CAM7-forward: 5′-CACACAACGGTC GAGAGACA-3′; human CEA CAM7-reverse: 5′- TTGGGTGGCTCCGAGAATAC-3′; human EPHB2-forward: 5′-GACCCTCCTTTTGAGTGGGG-3′; human EPHB2-reverse: 5′-GAGTTTGCAGCAACACCCTG-3′.

### 2.5 Drug therapy and analysis

Organoid cultures were passaged 2 days prior to drug treatment. For drug therapy, organoid cultures were washed with PBS and fresh media containing full supplementation and different concentrations of chemotherapy agents was added. To assess chemotherapy resistance, cultures were pre-treated with chemotherapy agents for 4 days followed by 3 days of fresh media without any selective conditions. This process was repeated two times. To minimize growth differences caused by initial organoid size, organoids were only selected for time-lapse imaging if their longest length was between approximately 200–300 μM. Time-lapse imaging was performed on a Nikon Eclipse Ti-E inverted microscope system (Nikon, UK) and images were captured every 6 h for 60 h. At the completion of imaging, images were converted to TIF files and the area of organoids was measured using in-house software written in MATLAB R2015b software. Growth curves were generated by comparing the area of organoids to their starting size and data is represented as the percentage of growth from time point 0. Apoptosis analysis was measured following 6 days of chemotherapy treatment by staining with the eBioscience™ Annexin V Detection Kit APC in accordance with the manufacturer guidelines (Thermo-Fisher; 88-8007-72).

### 2.6 Statistical analysis

All data is expressed as mean ± SEM and statistical analysis was performed by one-way analysis of variance (ANOVA) and the appropriate parametric (student *t*-test) statistical test using Sigmastat (Jandel Scientific). For QPCR analysis, p-values were derived from comparison between organoid cultures and the corresponding tumor biopsy. For all organoid growth analysis, p-values were generated from comparing untreated organoids with treatment groups. p-Values ≤ 0.05 were considered statistically significant.

## 3 Results

### 3.1 Organoids can be rapidly isolated from human CRC liver metastases and recapitulate human disease

**Fig. 1:**
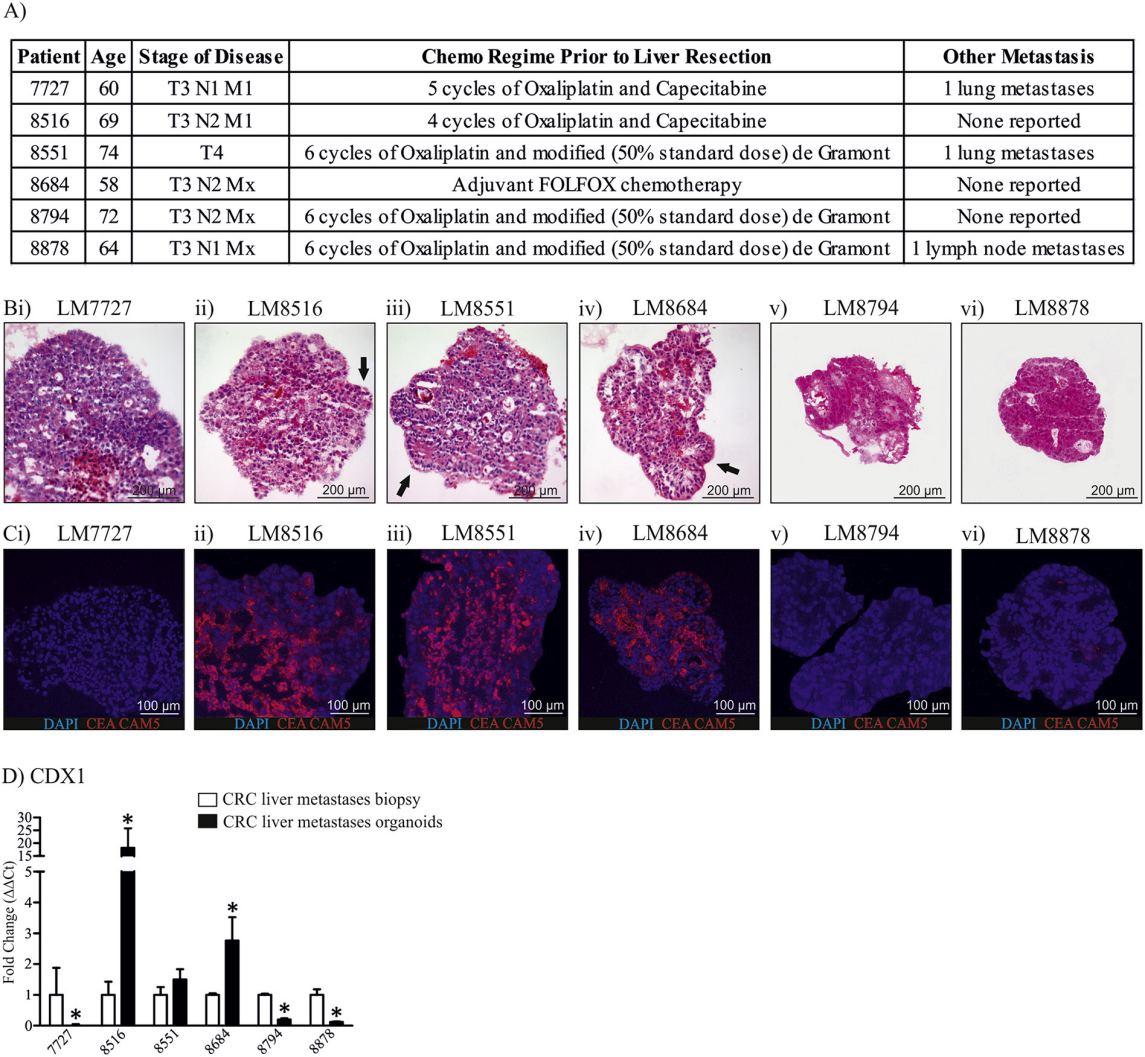
CRC liver metastases patient information and characterisation of organoid differentiation status. A) Patient information for 6 established CRC liver metastases organoids. B) H&E images of CRC liver metastases organoids. C) CEA CAM5 immunofluorescence staining in CRC liver metastases organoids. D) CDX1 mRNA expression in CRC liver metastases organoids. H&E images were taken at 10 × magnification, immunofluorescence images were taken at 20 × magnification. *Indicates statistically significant, p ≤ 0.05.

We previously developed a method for rapid culturing of CRC organoids using mechanical dissociation (Ashley et al., 2014). We therefore wanted to determine whether a similar method could be applied to human CRC liver metastases. Tissue collection was performed following patient consent. At the time of resection, all patients had progressed to an advanced stage (T3 or T4) and had received 3–6 cycles of adjuvant chemotherapy prior to resection (Fig. 1A). Two patients had lung metastases and one patient had lymph node metastases in addition to liver metastases (Fig. 1A).

Organoids were cultivated from CRC liver metastases pathological specimens. To generate organoids, pathological specimens were cut into 1 mm^3^ pieces, then mechanically dissociated by vigorously shaking in media with glass beads. The supernatant was collected and the process was repeated. The supernatant contained clumps of cells with complex, crypt-like structures and single cells as previously described (Ashley et al., 2014). Complex structures were then plated with or without Matrigel, and were cultured with full supplementation (Supp. Table 1). We collected 17 human CRC liver metastasis samples, 13 of which were successfully cultured as organoids. 3 cultures were unable to be passaged after thawing frozen stocks, and 2 cultures were lost to infection.

CRC and subsequent metastases can be graded according to differentiation status, whereby the more poorly differentiated, the more aggressive the tumor (Gleason and Mellinger, 1974). Therefore, we wanted to determine the differentiation status of the organoids. To assess the differentiation status of organoids, organoids were isolated and H&E stained (Fig. 1B). H&E staining showed LM8516, LM8551 and LM8684 to have lumen structures (arrows, Fig. 1B). To further assess organoid differentiation, we assessed the expression of the differentiation marker, CEA CAM5 by immunofluorescence (Fig. 1C). Consistent with H&E staining, LM8516, LM8851 and LM8684 organoids showed strong CEA CAM5 staining, whereas LM7727, LM8794 and LM8878 had minimal or no CEA CAM5 staining (Fig. 1C). We then assessed CDX1 mRNA expression between organoid cultures and their corresponding biopsy (Fig. 1D). CDX1 is a well described marker of colon differentiation and therefore it would be expected that well differentiated cultures would have increased CDX1 mRNA expression (Soubeyran et al., 1999). LM7727, LM8794 and LM8878 showed a decrease in CDX1 mRNA consistent with CEA CAM5 staining (Fig. 1D). In contrast, LM8516, LM8551 and LM8684, cultures which showed strong CEA CAM5 staining, had an increase in CDX1 mRNA expression relative to their respective biopsy samples, however the increase in CDX1 expression was not significant in LM8551 (Fig. 1D).

**Fig. 2:**
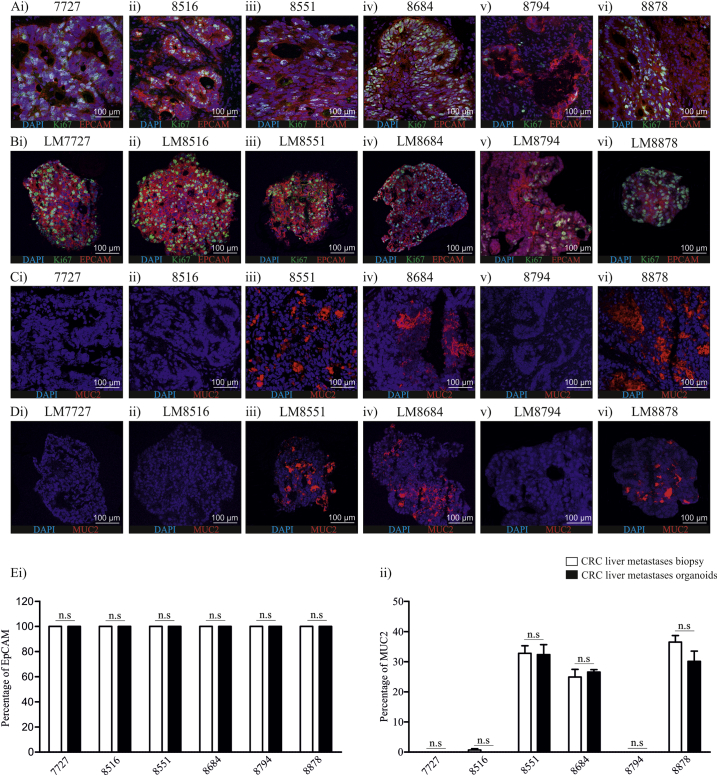
Comparison of EPCAM, Ki67 and MUC2 expression in CRC liver metastases pathological specimens and organoids using immunofluorescence. A) EPCAM and Ki67 expression in CRC liver metastases pathological specimens; B) EPCAM and Ki67 expression in CRC liver metastases organoids. C) MUC2 expression in CRC liver metastases pathological specimens. D) MUC2 expression in CRC liver metastases organoids. E) Quantification of i) EpCAM and ii) MUC2 in CRC liver metastases pathological specimens and CRC liver metastases organoids. All images were taken at 20 × magnification.

To validate that organoids contained progenitor cells and represent human disease, we assessed the expression of EpCAM and MUC2 in human pathological specimens and organoids by immunofluorescence (Fig. 2A–D). The proliferation mark, Ki-67 was also used. EpCAM is an antigen expressed by progenitor cells in multiple carcinomas (Baeuerle and Gires, 2007, Ricci-Vitiani et al., 2007, Yamashita et al., 2009), while MUC2 is a mucin expressed by normal colon epithelia, but differentially expressed in adenocarcinomas (Ajioka et al., 1996, Bu et al., 2010). All human pathological specimens and organoid cultures showed global expression of EpCAM and positive Ki-67 staining (Fig. 2A & B). The expression pattern of Ki-67 was also similar between human pathological specimens and CRC liver metastases organoids (Fig. 2A & B). In adenocarcinoma, MUC2 expression inversely correlates to disease severity (Lugli et al., 2007), and reduced MUC2 is associated with advanced stages of disease (Lugli et al., 2007). Therefore, we wanted to assess the expression of MUC2 in human pathological specimens and CRC liver metastases organoids (Fig. 2C–E). MUC2 was absent in LM7727, LM8516 and LM8794 human pathological specimens and organoids (Fig. 2C & D). In contrast, LM8551, LM8684 and LM8878 had positive MUC2 staining in human pathological specimens and this corresponded to positive MUC2 staining in LM8551, LM8684 and LM8878 organoids (Fig. 2C & D). We then wanted to determine whether the amount of EpCAM and MUC2 positive staining was similar between pathological specimens and organoids (Fig. 2E). Quantification of EpCAM and MUC2 showed no significant difference between pathological specimens and corresponding organoids (Fig. 2E). Collectively, these data suggest that rapidly cultured CRC liver metastases organoids maintain some morphological characteristics of their corresponding tumor pathological specimens.

### 3.2 Organoids can be cultured without Matrigel and can proliferate without supplementation

**Supplementary Fig. 1:**
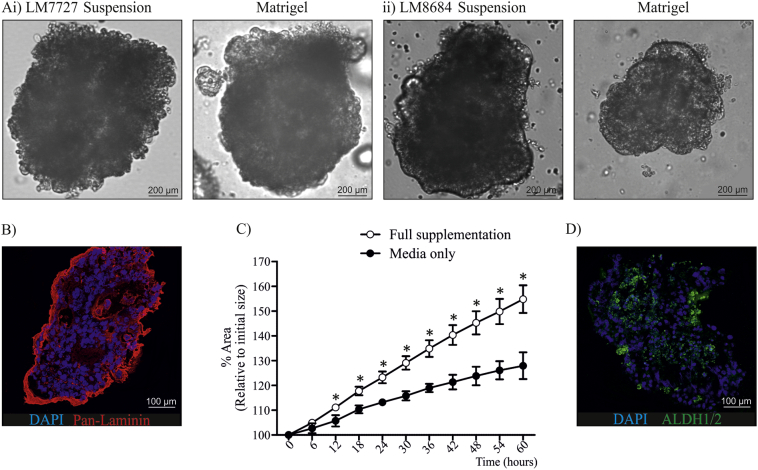
Comparison of organoids grown with and without Matrigel, and the effect of removing growth factor supplementation. A) Morphological comparison of i) LM7727 and ii) LM8684 cultured in suspension or in Matrigel, 10 × magnification. B) Expression of the extracellular matrix protein, Pan-Laminin in organoid LM7727, 20 × magnification C) Comparison of organoid growth percentage with and without growth factor supplementation. D) Expression of the ALDH1/2 in organoid LM8684, 20 × magnification.*Indicates statistically significant, p ≤ 0.05.

After we had established that organoids were morphologically similar to their corresponding tumor pathological specimens we wanted to determine whether culturing organoids in suspension compared to Matrigel altered morphology (Sup. Fig. 1A & B), and if growth factor supplementation sufficiently influenced growth of established organoids (Sup. Fig. 1C). We observed that organoids were remarkably similar when grown with or without Matrigel (Sup. Fig. 1A), suggesting they produce sufficient extracellular matrix (ECM) proteins to maintain their complex structure. To confirm this, we stained organoids with the ECM protein, pan-laminin (Sup. Fig. 1B). As expected, pan-laminin was predominately expressed at the border of organoids (Sup. Fig. 1B), confirming that organoids express and secrete ECM proteins allowing them to maintain a complex structure in suspension. To assess how growth factor supplementation influenced growth of established organoids, organoids were cultured in Matrigel with or without supplementation and time-lapse images were captured for 60 h (Sup. Fig. 1C). Culturing organoids without supplementation severely delayed growth (Sup. Fig. 1C), with a significant growth difference observed only 12 h following the removal of additives (Sup. Fig. 1C). However, despite the growth delay, we did not observe a morphological difference during the course of imaging.

### 3.3 CRC liver metastases organoids show characteristic of colonic origin and differential expression of stem cell markers

**Fig. 3:**
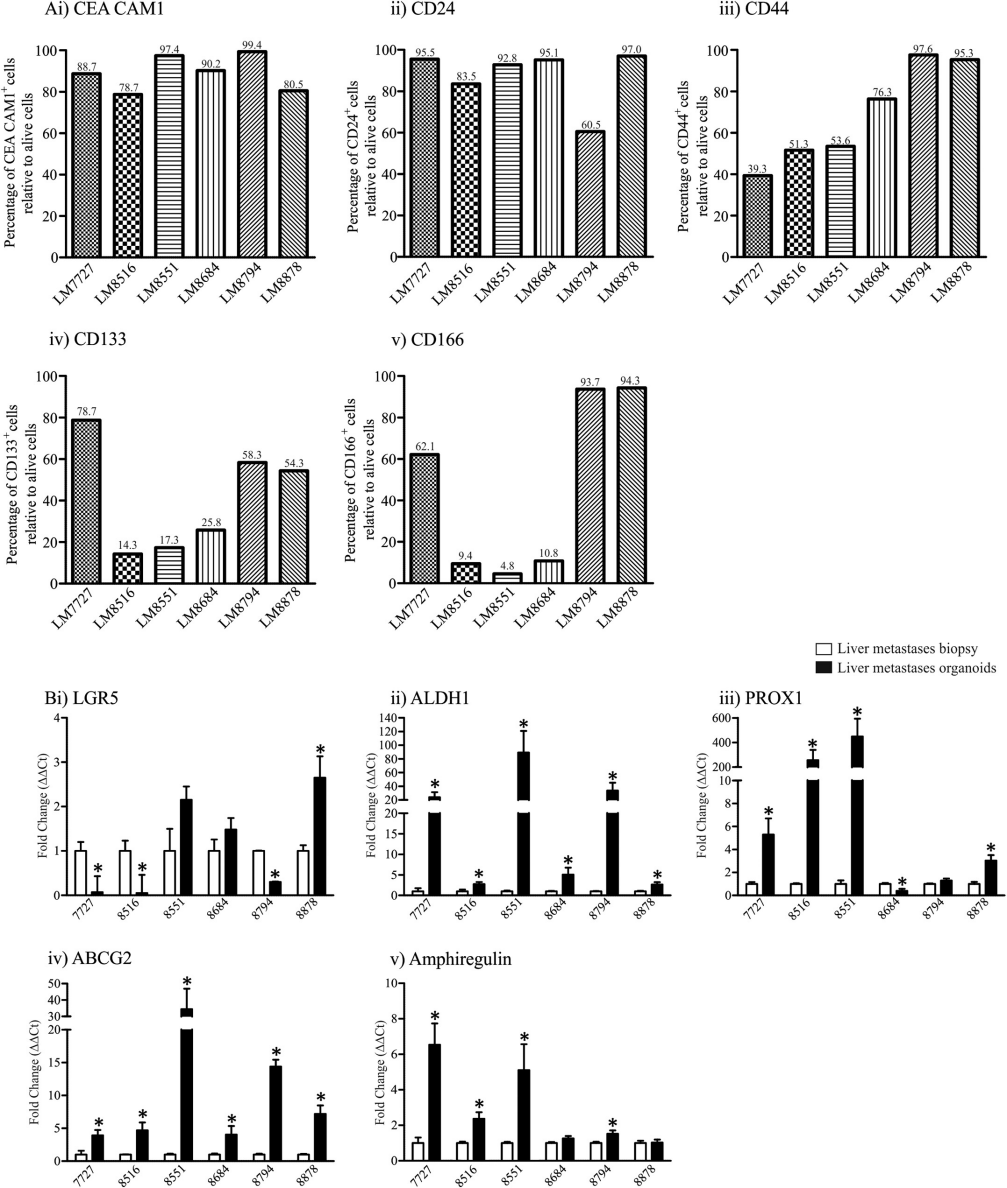
Flow cytometry and mRNA analysis of stem cell markers in CRC liver metastases organoids. A) Flow cytometry analysis of i) CEA CAM1; ii) CD24; iii) CD44; iv) CD133 and v) CD166. B) QPCR analysis of i) LGR5; ii) ALDH1; iii) PROX1, iv) ABCG2 and v) Amphiregulin. *Indicates statistically significant, p ≤ 0.05.

**Supplementary Fig. 2:**
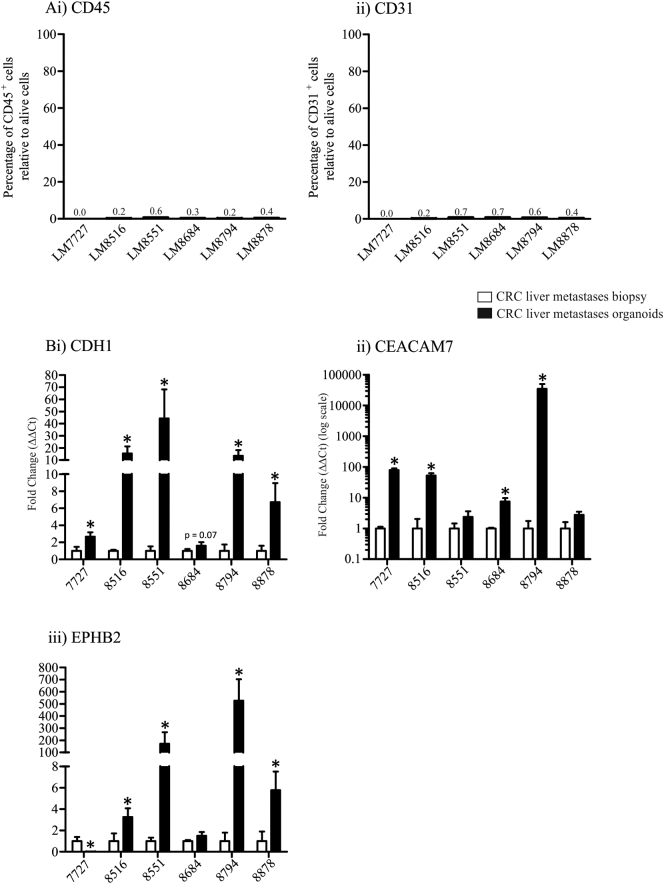
A) Flow cytometry analysis of i) CD45 and ii) CD31 in CRC liver metastases organoids. B) QPCR analysis of epithelial markers in CRC liver metastases organoids compared to corresponding tumor biopsies; i) CDH1, ii) CEACAM7 and iii) EPHB2. *Indicates statistically significant, p ≤ 0.05.

Emerging evidence suggests CRC contain cancer stem cells. Cancer stem cells are a niche population of cells within malignancies which maintain the capacity to self-renew and are resistant to chemotherapy and irradiation (Sosa et al., 2014). CRC stem cells can express various surface markers, therefore we examined whether CRC liver metastases organoids derived using rapid dissociation expressed the well described stem cell surface markers, CD24, CD44, CD133 and CD166, as well as the known adenocarcinoma biomarker, CEA CAM1 by flow cytometry. CEA CAM1, was highly expressed in all organoids (78.7–97.4%, Fig. 3Ai) confirming organoids were derived from adenocarcinoma tissues. Interestingly, of the stem cell surface markers we assessed by flow cytometry, only CD24 was abundantly expressed in all cultures (60.5–95.5%, Fig. 3Aii), while CD44 showed more variable expression (39.3–97.6%, Fig. 3iii). For both CD133 and CD166, substantial positive staining by flow cytometry was only observed for LM7727, LM8794 and LM8878 organoids (Fig. 3iv & v), cultures which were less differentiated according to CEA CAM5 and CDX1 expression (Fig. 1). For LM7727, LM8794 and LM8878 organoids, CD133 and CD166 was expressed by 54.3–78.7% and 62.1–94.3% of cells respectively, whereas the well differentiated organoids (LM8516, LM8551 and LM8684) only expressed CD133 and CD166 between 14.3 and 25.8% and 4.5–10.8% respectively (Fig. 3iv & v). We also tested the immune cell surface marker, CD45 and the endothelial surface marker, CD31 by flow cytometry and did not observe any positive staining (Sup. Fig. 2A), confirming the epithelial origin of the CRC liver metastases organoids, and the absence of contamination with other cell types.

Once we had established that liver metastases organoids express stem cell surface markers, we wanted to determine whether they showed an enrichment for stem cell genes compared to their corresponding biopsy. Therefore, we assessed the mRNA expression of well described stem cell genes, including ALDH1, PROX1 and LGR5, as well as ABCG2, a stem cell gene associated with chemotherapy resistance (Stacy et al., 2013), and Amphiregulin, a gene shown to be important in self-renewal (Booth et al., 2010). Gene expression was assessed by PCR analysis; organoids and corresponding human pathological specimens were prepared in quadruplet. Surprisingly, LGR5 showed no expression pattern between organoids (Fig. 3Bi). In contrast, compared to their respective pathological specimens, all organoids showed a significant increase in ALDH1 mRNA expression, however at varied degrees (Fig. 3Bii), and this was confirmed by immunofluorescence staining (Sup. Fig. 1D). Similarly, PROX1 was increased in all cultures besides LM8684 (Fig. 3Biii), and ABCG2 was significantly increased in all cultures (Fig. 3Biv). The chemotherapy resistant gene, Amphiregulin was increased in 4 cultures and unaltered in the other 2 (Fig. 3Bv). To determine whether the enrichment in stem cell genes in organoid cultures was due to an increase in epithelial cells (at the expense of stroma, endothelial cells and immune cells), we analysed the expression of 3 epithelial markers, CDH1, CEACAM7 and EPHB2 in human pathological specimens and organoid cultures (Sup. Fig. 2B). CDH1 and CEACAM7 was increased in all organoid cultures compared to their corresponding biopsy, while EPHB2 was increased in all cultures besides LM7727 (Sup. Fig. 2B). Collectively, this suggests that increased expression of stem cell genes in organoid cultures may be due to an enrichment of epithelial cells.

### 3.4 CRC liver metastases organoids can be screened for susceptibility to novel drug therapies

**Fig. 4:**
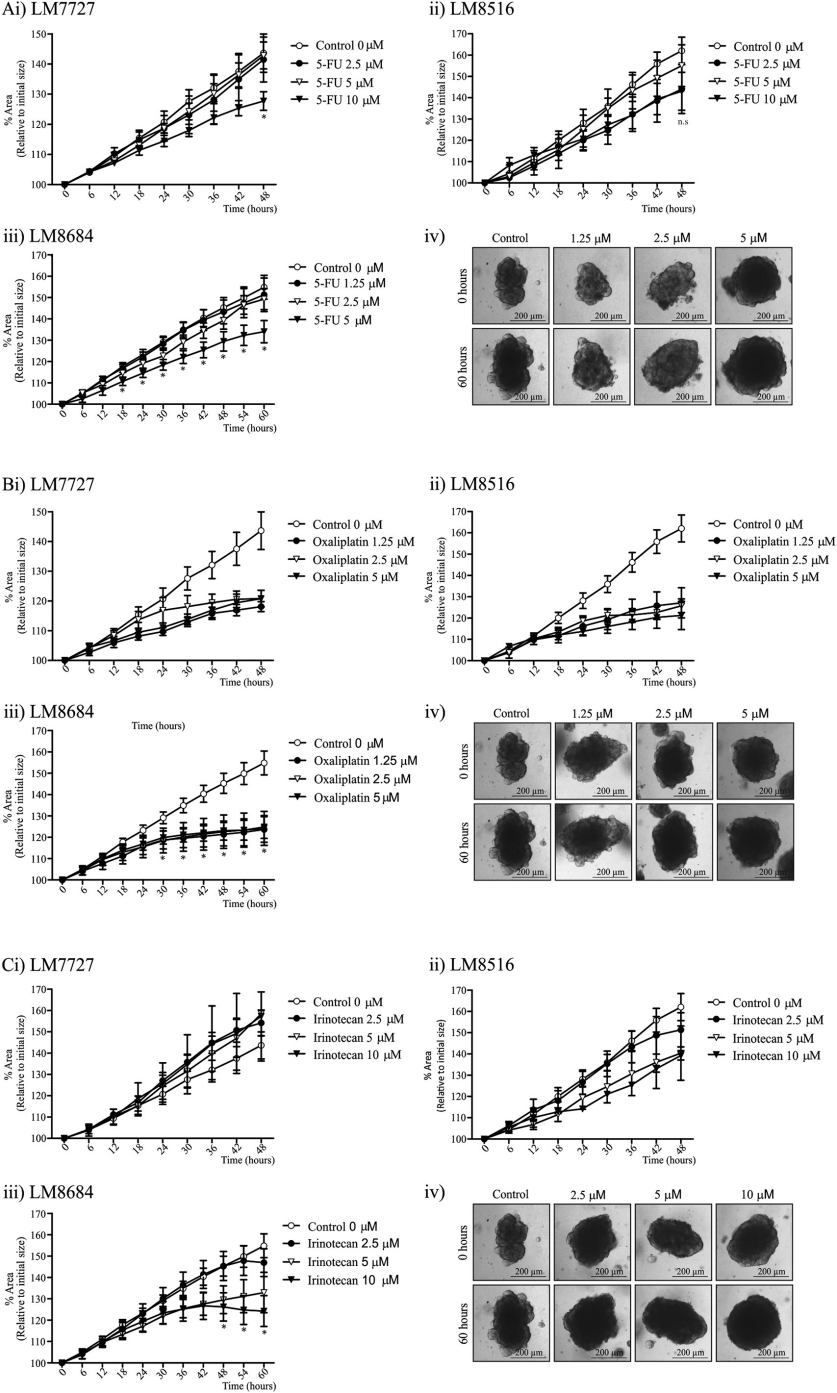
Time-lapsing imaging of organoid growth and response to drug therapies. A) Growth percentage and images of organoids following 1.25–10 μM 5-FU; i) Growth curves of LM7727, ii) Growth curves of LM8516, iii) Growth curves of LM8684 and iv) Representative images of LM8684 following 5-FU treatment. B) Growth percentage and images of organoids following 1.25–5 μM Oxaliplatin; i) Growth curves of LM7727, ii) Growth curves of LM8516, iii) Growth curves of LM8684 and iv) Representative images of LM8684 following Oxaliplatin treatment. C) Growth percentage and images of organoids following 2.5–10 μM Irinotecan; i) Growth curves of LM7727, ii) Growth curves of LM8516, iii) Growth curves of LM8684 and iv) Representative images of LM8684 following Irinotecan treatment. All images were taken at 4 × magnification. *Indicates statistically significant, p ≤ 0.05.

After we had established that CRC liver metastases organoids recapitulate some aspects of human disease, we wanted to determine whether cultures could be utilized for drug testing. To assess the effectiveness of drug therapies, LM7727, LM8516 and LM8684 organoids were grown in Matrigel. A 2-fold serial dilution of therapeutic drugs ranging from 0.625 μM to 10 μM was then added to the media and time-lapse images were captured for 60 h. Prior to liver resection and sample collection, all patients had received Oxaliplatin and Capecitabine (Fig. 1A). Oxaliplatin causes cross-linking of DNA leading to inhibition of DNA synthesis and transcription (Ehrsson et al., 2002). Capecitabine is converted to 5-Fluorouracil (5-FU). 5-FU predominately causes cell death by blocking the synthesis of thymidine, consequently inhibiting DNA replication (Longley et al., 2003). Therefore, we first wanted to test whether these therapies were still effective on patient-derived CRC liver metastases organoids, which had previously received treatment (Fig. 4A & B). For LM7727, 10 μM 5-FU was required to cause growth delay, whereas, LM8516 showed no significant growth delays following 2.5–10 μM 5-FU treatment (Fig. 4Ai & ii), and 5-FU delayed LM8684 organoid growth at a concentration of 5 μM (Fig. 4Aiii & iv). For LM8684, a significant growth difference was observed 18 h post-treatment, however despite growth delay, there were no signs of cell death in cultures at the completion of imaging (Fig. 4Aiv). In contrast to 5-FU, Oxaliplatin caused a similar growth arrest in LM7727, LM8516 and LM8684 organoids at all concentrations tested (1.25 μM, 2.5 μM and 5 μM; Fig. 4B). 0.625 μM Oxaliplatin also caused a similar growth arrest in organoids. After establishing the susceptibility of organoids to 5-FU and Oxaliplatin treatment, we wanted to assess whether Irinotecan, a chemotherapy agent commonly used in combinations with other drug therapies (Tournigand et al., 2004), was effective at inhibiting organoid growth (Fig. 4C). In addition, patients whose pathological specimens were used to culture CRC liver metastases organoids had not received Irinotecan therapy (Fig. 1A). LM7727 and LM8516 organoids did not show any significant growth delay in response to 2.5–10 μM Irinotecan for the time-lapse imaging. In contrast, following 10 μM Irinotecan treatment, LM8684 organoid size began to decline 42 h post-treatment and continued to decline for the duration of the imaging, and a statistically significant growth difference was observed 48 h post-treatment (Fig. 4Ciii & iv). In addition, 10 μM Irinotecan caused significant cell death. Morphologically, organoids began to loss structural integrity around 42 h and by 60 h became dense, compact and consisted of apoptotic-like cells (Fig. 4Ciii & iv).

**Fig. 5:**
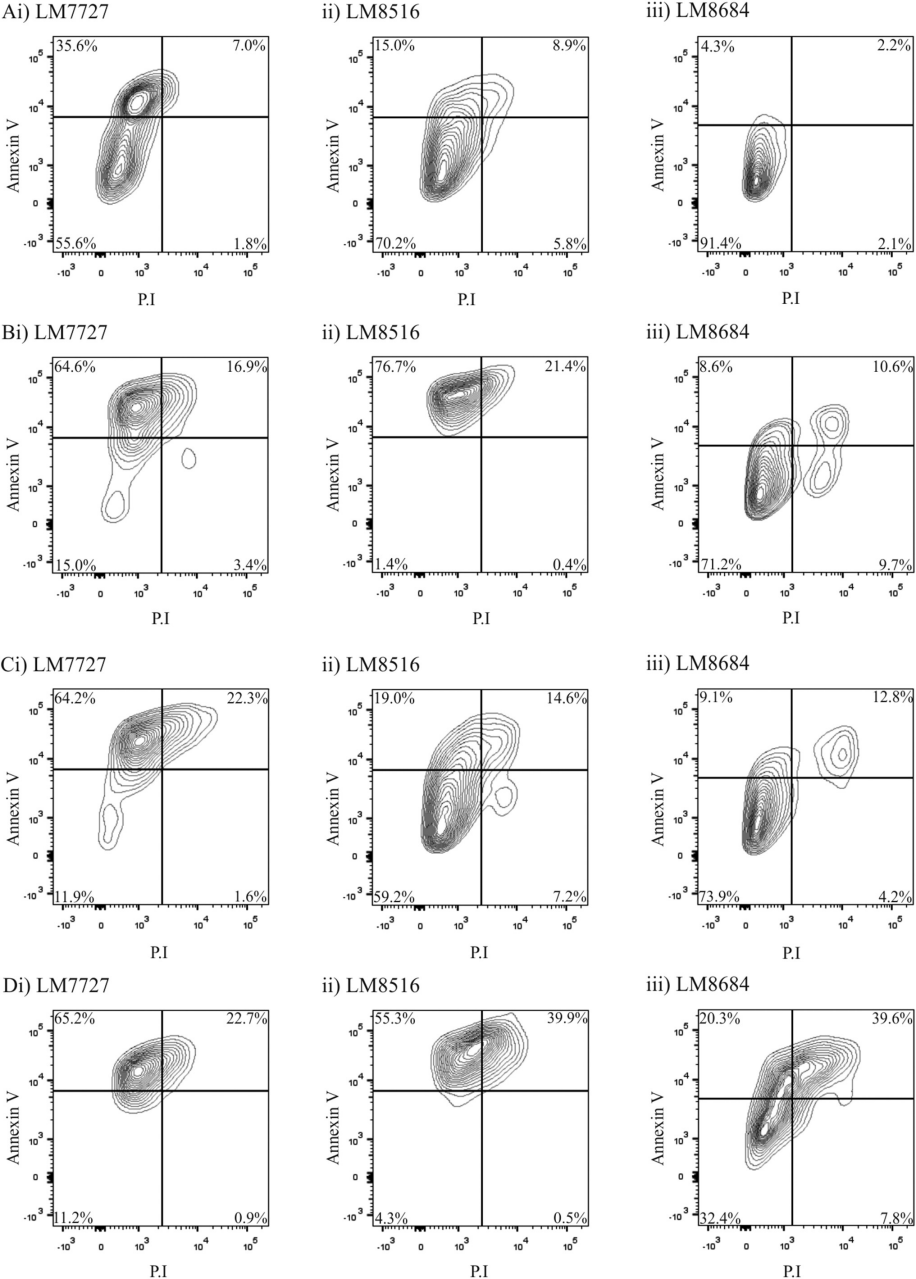
Flow cytometry analysis of Annexin V and P·I following 6 days of chemotherapy. A) Flow cytometry plots of untreated (control) organoids; i) LM7727, ii) LM8516 and iii) LM8684. B) Flow cytometry plots of organoids following 6 days of 5 μM 5-FU; i) LM7727, ii) LM8516 and iii) LM8684. C) Flow cytometry plots of organoids following 6 days of 1.25 μM Oxaliplatin; i) LM7727, ii) LM8516 and iii) LM8684. D) Flow cytometry plots of organoids following 6 days of 10 μM Irinotecan; i) LM7727, ii) LM8516 and iii) LM8684.

Chemotherapy can take several days to induce tumor apoptosis, however we were unable to time-lapse image for that duration because organoids required fresh media every 3 days. Therefore, we treated LM7727, LM8516 and LM8684 organoid cultures with 5 μM 5-FU, 1.25 μM Oxaliplatin or 10 μM Irinotecan for 6 days, and then assessed the expression of the apoptotic marker, Annexin V and P·I by flow cytometry analysis (Fig. 5). Annexin V^+^ P.I^−^ indicates early apoptosis, Annexin V^+^ P.I^+^ indicates late apoptosis and Annexin V^−^ P.I^+^ indicates cell death. Control LM7727, LM8516 and LM8684 organoid cultures, showed similar staining for Annexin and P.I (Fig. 5A), with cells either not undergoing any apoptosis (Annexin V^−^ P.I^−^), or early apoptosis (Fig. 5A). Following 6 days of 5 μM 5-FU, LM7727 and LM8516 showed an increase in early apoptotic cells (Fig. 5Bi & ii). In contrast, LM8684 had a greater shift towards late apoptosis and cell death (Fig. 5Biii). This data is consistent with time-lapse imaging, where LM8684 was more susceptible to 5-FU treatment (Fig. 4A). LM7727 had a large shift towards early apoptosis in response to Oxaliplatin treatment (Fig. 5Ci), while LM8516 and LM8684 were less affected (Fig. 5Cii & iii), despite all cultures having delayed growth in response to Oxaliplatin treatment during time-lapse imaging (Fig. 4B). This suggests that, consistent with its known function to inhibit DNA synthesis, Oxaliplatin limits tumor growth through inhibiting proliferation, not by inducing apoptosis. Finally, Irinotecan treatment induced a large shift towards early and late apoptosis in all cultures (Fig. 5C), despite only observing a significant decrease in organoids growth in LM8684 (Fig. 4C). Collectively, these data demonstrate that organoids are susceptible to different chemotherapy agents, and have the potential to be utilized as surrogates for drug screening.

### 3.5 CRC liver metastases organoids show growth delay, but not death in response to chemotherapy

**Fig. 6:**
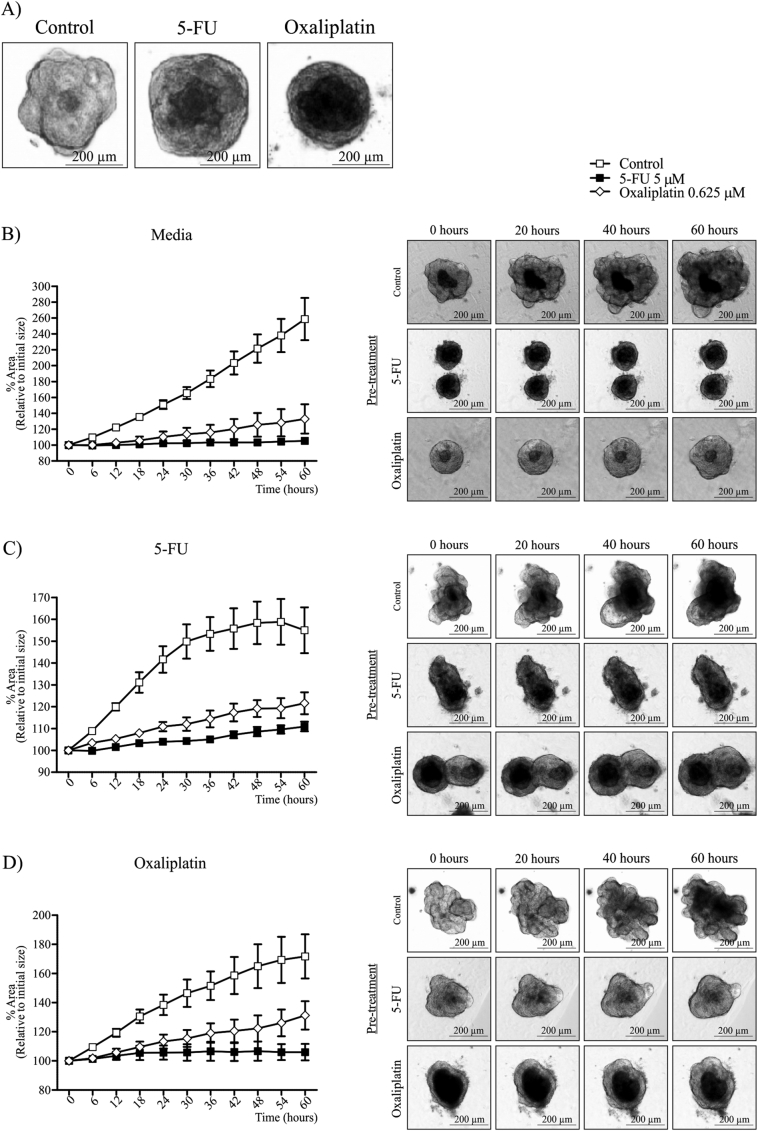
Time-lapsing imaging of organoid growth following pre-treatment of 5-FU and Oxaliplatin. A) Organoid morphology following 3 rounds of either 5 μM 5-FU or 0.625 μM Oxaliplatin treatment. Organoid growth percentage in (B) untreated media, (C) 10 μM 5-FU and (D) 0.625 μM Oxaliplatin after three rounds of 5-FU or Oxaliplatin treatment. All images were taken at 4 × magnification. *Indicates statistically significant, p ≤ 0.05.

We observed that CRC liver metastases organoids showed growth delay in response to 5-FU and Oxaliplatin treatment, however did not undergo significant cell death (Fig. 4, Fig. 5). Therefore, we wanted to determine whether multiple rounds of 5 μM 5-FU and 0.625 μM Oxaliplatin could induce chemotherapy resistance in organoid cultures (Fig. 6A–D). Cultures were seeded in Matrigel, and exposed to three rounds of chemotherapy, whereby chemotherapy agents were added to media for 4 days, followed by 3 days without chemotherapy supplementation. Organoid morphology was altered following 3 rounds of 5-FU or Oxaliplatin, most noticeably was the loss of distinct lumen-like structures (Fig. 6A). We then wanted to test whether our chemotherapy regimen affected growth in untreated media (Fig. 6B). Untreated organoids showed a consistent growth pattern as previously observed (Fig. 6B). However, pre-treatment with 5-FU or Oxaliplatin severely delayed organoid growth (Fig. 6B). This was particularly evident following 5-FU treatment, with organoids growing only 6% over 60 h (Fig. 6B). Despite this growth pattern, organoids showed no signs of cell death (Fig. 6B).

**Supplementary Fig. 3:**
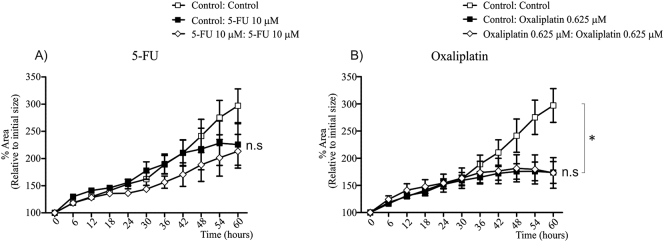
Time-lapsing imaging of organoid growth following one round of pre-treatment of 5-FU and Oxaliplatin. A) 5-FU and B) Oxaliplatin. *Indicates statistically significant, p ≤ 0.05.

To further assess the effect of chemotherapy pre-treatment, organoids were exposed to 5-FU or Oxaliplatin for 60 h (Fig. 6C & D). When exposed to 5-FU, untreated organoids expanded more rapidly than 5-FU and Oxaliplatin pre-treated organoids (Fig. 6C). However after 30 h, untreated organoids exposed to 5-FU showed a significant reduction in growth rate, and organoid size began to decline at 54 h (Fig. 6C). In contrast, 5-FU and Oxaliplatin pre-treated organoids showed no signs of growth delay following 5-FU treatment (Fig. 6C). A similar observation was observed following Oxaliplatin treatment (Fig. 6D). We also tested the effects of a single round of chemotherapy pre-treatment. Interestingly, one round of chemotherapy did not alter the growth patterns of organoid cultures (Supplemental Fig. 3). Collectively, these data suggests that multiple rounds of chemotherapy can cause CRC liver metastases organoids to acquire chemotherapy resistance, albeit at a significant reduction in growth rate.

## 4 Discussion

CRC liver metastasis is a major health burden, however investigating the mechanisms of liver metastasis remains challenging. In particular, chemotherapy treatment of CRC liver metastases is problematic and relapse is prevalent (Scheel and Weinberg, 2012). Here, we demonstrate that human CRC liver metastases can be rapidly cultured as organoids using mechanical dissociation, and these cultures can be used as surrogates for drug screening and acquire chemotherapy resistance, expanding on previous work done on establishing CRC organoids (Ashley et al., 2014, Ashley et al., 2013, Fujii et al., 2016, Sato et al., 2011, Weeber et al., 2015).

In our hands, culturing with Matrigel did not alter organoid morphology, however we did not investigate whether growth rates differed. Furthermore, a striking growth difference was observed between organoids cultured in media with full supplementation compared to no additives, however CRC liver metastases organoids were still expanding 60 h post-supplementation removal. This suggests that, once established, CRC liver metastases organoids are capable of surviving for, at least, a short period of time without supplementation. This is a similar observation to growth patterns of CRC organoids (Lee et al., 2015).

We used immunofluorescence and flow cytometry to confirm organoids derived from CRC liver metastases pathological specimens contained progenitor cells and originated from adenocarcinoma. All CRC liver metastases organoids showed global expression of EpCAM, a marker of progenitor cells in multiple carcinomas (Baeuerle and Gires, 2007, Ricci-Vitiani et al., 2007, Yamashita et al., 2009). Furthermore, CRC liver metastases organoids demonstrated high expression of the adenocarcinoma marker, CEA CAM1 by flow cytometry. Although early studies suggest CEA CAM1 acts as a tumor suppressor (Leung et al., 2006), more recent evidence strongly suggests that high CEA CAM1 expression in tumor cells directly correlates to poor prognosis and metastasis in many gastrointestinal cancers (Kang et al., 2007). The data presented here supports these recent findings, with CRC liver metastases organoids described here containing a large portion of tumor cells positive for CEA CAM1 (78.7–97.4%), which is substantially more than previously reported in CRC organoids (Lee et al., 2015). In contrast to CEA CAM1, loss of MUC2 expression correlates to poor prognosis in CRC patients (Ajioka et al., 1996), and depletion of MUC2 in mice causes spontaneous colitis (Van der Sluis et al., 2006). In our CRC liver metastases organoids, MUC2 expression was either completely absent or expressed in only 25–40% of cells. This is strikingly different to colonic organoids which express MUC2 globally when cultured with specific growth factors (Sato et al., 2011). The expression pattern of EpCAM, CEA CAM1 and MUC2 in CRC liver metastases organoids demonstrates that CRC liver metastases organoids show characteristics of advanced stages of disease.

Recent evidence suggests that a population of cancer cells, known as cancer stem cells, have the self-renewal and differentiation capacity to form new tumor populations (Beck and Blanpain, 2013). While these cells may not necessarily arise from normal stem cells, their unique properties makes them resistance to conventional chemotherapy agents and irradiation (Hong et al., 2009, Touil et al., 2014). Cancer stem cells can be identified by various surface markers however, such markers can be variably expressed between cancer populations (Medema, 2013). Furthermore, specific genes have been characterized as being predominately expressed by cancer stem cells (Medema, 2013). While CD24 and CD44 were expressed at relatively similar proportions by CRC liver metastases organoids, CD133 and CD166 were only abundantly expressed by LM7727, LM8794 and LM8878, cultures which showed poor expression of the differentiation marker, CEA CAM5 by immunofluorescence, and had decreased mRNA expression of the differentiation marker, CDX1 when compared to patient samples. It has recently been demonstrated that CD133 suppresses differentiation in neuroblastoma cells (Takenobu et al., 2011), therefore the high proportion of CD133^+^ cells in LM7727, LM8794 and LM8878 may account, in part, for the poorly differentiated status in these cultures. Furthermore, CD133 is a predictor of poor patient outcome in CRC (Reggiani Bonetti et al., 2012), similar to poor differentiation status (Gleason and Mellinger, 1974). Likewise, differentiation of stromal cells has been shown to cause downregulation of CD166 (Walmsley et al., 2015), and high CD166 expression correlates to poor disease free survival of rectal cancer patients (Sim et al., 2014). Collectively, our data supports findings that CD133 and CD166 are highly expressed in poorly differentiated cells.

Genes specifically expressed by cancer stem cells influence the severity of CRC and subsequent metastases (Wan et al., 2013). We analysed cancer stem cell genes in CRC liver metastases organoids known to correlate with disease progression and chemotherapy resistance. ALDH1 correlates with disease stage (Chen et al., 2015), PROX1 drives metastasis (Ragusa et al., 2014) and LGR5 and ABCG2 are associated with chemoresistance in patients (Stacy et al., 2013, Liu et al., 2013). We observed a general increase in stem cell genes in organoid cultures compared to pathological specimens. This was not surprising considering organoid cultures are of epithelial origin as demonstrated by EpCAM and CEACAM1 positively, whereas pathological specimens contain fibroblasts/stellate cells, immune cells and blood vessels. In support of this, organoid cultures had a similar increase in epithelial markers compared to their corresponding pathological specimens, and did not contain CD45 or CD31 cells. The correlation between epithelial markers and stem cell genes suggests that the enrichment of epithelial cells during organoid culturing may lead to increased expression of stem cell genes in organoid cultures compared to pathological specimens.

Finally, we demonstrate that CRC liver metastases organoids can be effectively utilized for drug screening and can acquire chemotherapy resistance. Following multiple rounds of chemotherapy, CRC liver metastases organoids showed an altered morphology and their growth was severely hampered. Furthermore, we demonstrate that CRC liver metastases organoids acquire chemotherapy resistance to 5-FU and Oxaliplatin, consist with previous findings (Fillmore and Kuperwasser, 2008). Such drugs predominately target proliferating cells (Ehrsson et al., 2002, Longley et al., 2003), therefore it is not surprising that the significant growth delay in CRC liver metastases organoids observed following multiple rounds of chemotherapy permits chemotherapy evasion.

Here, we demonstrate that rapidly derived CRC liver metastases recapitulate some aspects of human disease. CRC liver metastases organoids can be used for evaluating drug response and chemotherapy resistance in advanced stages of disease. Furthermore, using such cultures, it may be possible to compare the effectiveness of novel drug therapies at early and advanced stages of disease.

The following are the supplementary data related to this article.

Supplementary Table 1 Growth factors added to Advanced DMEM/F12 + GlutaMAX when culturing organoids from human liver metastases biopsies.
Supplementary Table 2: Fluorochromes used to assess the expression of stem cell markers in CRC liver metastases organoids. Supplementary Table 1

## Author contribution

Jon N Buzzelli: Conception and design, Collection and assembly of data, Data analysis and interpretation, Manuscript writing; Djamila Ouaret: Conception and design, Collection and assembly of data; Graham Brown: Administrative support; Philip D. Allen: Administrative support; Ruth J Muschel: Conception and design, Financial support, Manuscript writing, Final approval of manuscript.

## Statement

The authors declare no conflicts of interest

## Funding

Cancer Research UK; H3RWHC00, H302.1.
